# Exploring an alternative explanation for the second phase of viral decay: Infection of short-lived cells in a drug-limited compartment during HAART

**DOI:** 10.1371/journal.pone.0198090

**Published:** 2018-07-17

**Authors:** Steven Sanche, Thibault Mesplède, Nancy L. Sheehan, Jun Li, Fahima Nekka

**Affiliations:** 1 Faculté de Pharmacie, Université de Montréal, Montreal, Quebec, Canada; 2 McGill University AIDS Centre, Lady Davis Institute for Medical Research, Jewish General Hospital, Montreal, Quebec, Canada; 3 Department of Microbiology and Immunology, Faculty of Medicine, McGill University, Montreal, Quebec, Canada; 4 Chronic Viral Illness Service, McGill University Health Centre, Montreal, Quebec, Canada; 5 Centre de recherches mathématiques, Université de Montréal, Montreal, Quebec, Canada; University of Pittsburgh Centre for Vaccine Research, UNITED STATES

## Abstract

Most HIV-infected patients who initiate combination antiretroviral therapy experience a viral load decline in several phases. These phases are characterized by different rates of viral load decay that decrease when transitioning from one phase to the next. There is no consensus as to the origin of these phases. One hypothesis put forward is that short- and long-lived infected cells are responsible for the first and second phases of decay, respectively. However, significant differences in drug concentrations are observed in monocytes from various tissues, suggesting the first two phases of decay in viral loads could instead be attributed to short-lived cells being differently exposed to drugs. Compared to a well-exposed compartment, new cell infection can be expected in a compartment with limited drug exposure, thus leading to a slower viral load decay with potential virologic failure and drug resistance. In the current study, the latter hypothesis was investigated using a model of viral kinetics. Empirical datasets were involved in model elaboration and parameter estimation. In particular, susceptibility assay data was used for an *in vitro* to *in vivo* extrapolation based on the expected drug concentrations inside physiological compartments. Results from numerical experiments of the short-term evolution of viral loads can reproduce the first two phases of viral decay when allowing new short-lived cell infections in an unidentified drug-limited compartment. Model long-term predictions are however less consistent with clinical observations. For the hypothesis to hold, efavirenz, tenofovir and emtricitabine drug exposure in the drug-limited compartment would have to be very low compared to exposure in peripheral blood. This would lead to significant long-term viral growth and the frequent development of resistant strains, a prediction not supported by clinical observations. This suggests that the existence of a drug-limited anatomical compartment is unlikely, by itself, to explain the second phase of viral load decay.

## Introduction

Viral loads in the plasma of patients initiating highly active antiretroviral therapy (HAART) generally decrease very rapidly during the first days of treatment before reaching a slower second phase of decay.[1, 2] In fact, up to four phases of decreasing viral load can be observed, each new phase being slower than the previous one.[3] These phases are the result of the complex interaction between host, drugs and virus. The existence of multiple phases of viral decay challenges our understanding of this interaction.[4]

**Fig 1:**
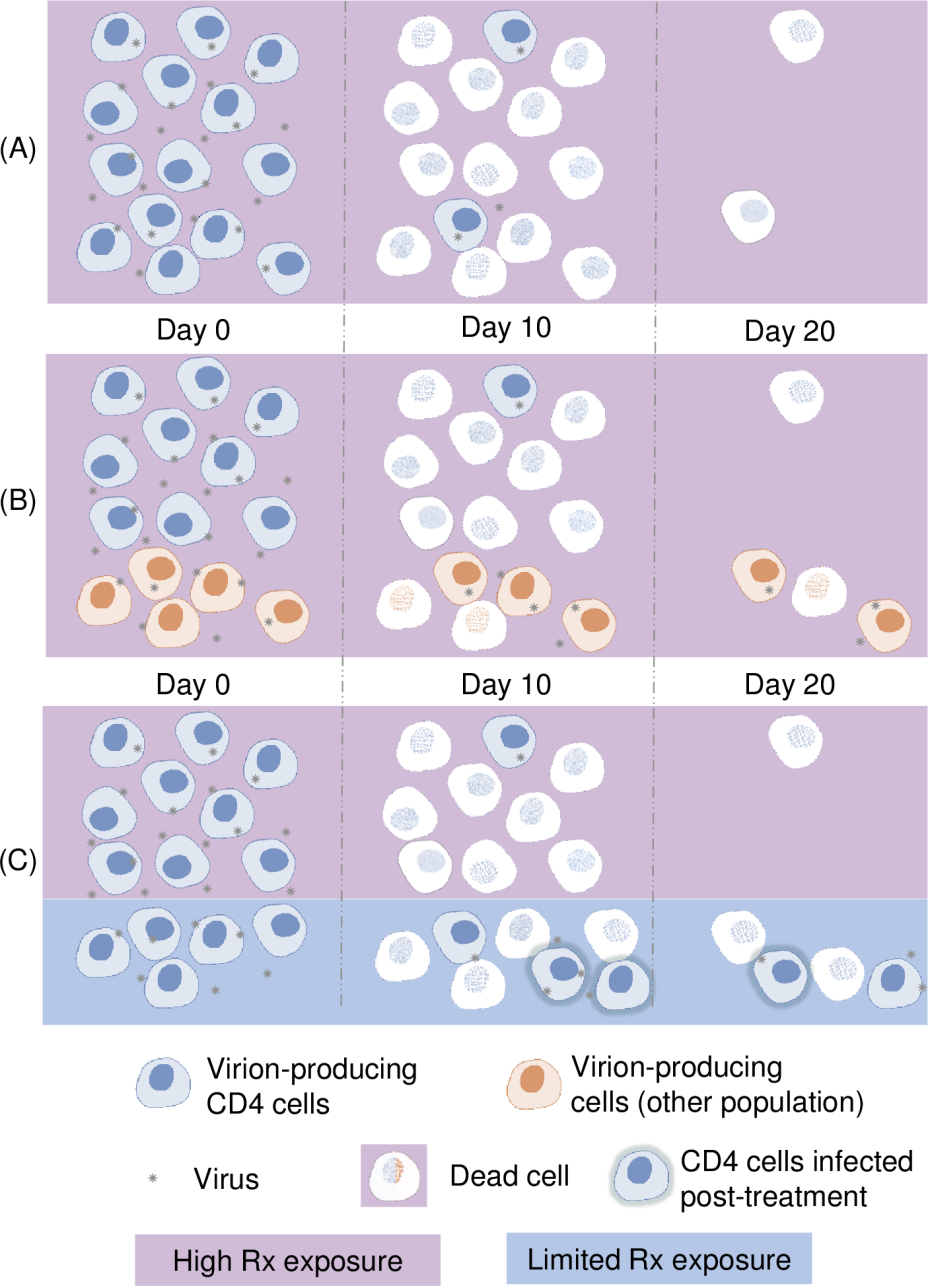
Hypotheses related to the decay of virion-producing cells after treatment initiation. A) Virion-producing cells are short-lived infected CD4 cells and all new cell infections are prevented due to high drug exposure. This translates into only one phase of viral decay; B) There are two types of virion-producing cells having half-lives of around 1 (short-lived) and 14 days (long-lived), respectively, and all new cell infections are prevented due to high drug exposure.[1] This translated into two phases of viral decay C) Virion-producing cells are mostly short-lived infected CD4 cells located in two compartments, one with high (pink) and one with low (blue) drug exposure. The compartment with low drug exposure partially allows new cell infections, effectively leading to two phases of viral decay. The compartment is not associated to a specific tissue at this point, as its existence is hypothesized.

In the following, we will demonstrate that there are multiple rational explanations for the first two phases of viral load decay. First, we will infer that a set of three assumptions is inconsistent with multiple phases of viral decay. Under the first assumption, viral loads during the first and second phases of viral decay mainly come from one infected cell population: CD4 cells having a half-life of virion production of about one day (short-lived). Under the second assumption, viral loads are proportional to the number of infected cells. This assumption is partially supported by results suggesting rapid virion clearance in lymphoid tissue and plasma (no accumulation of virions).[5, 6] Under the third assumption, HAART has the capacity to fully inhibit all new cell infections. If all of these assumptions were true, there would be only one phase of viral decay, as depicted by Fig 1A. Indeed, under assumption one and two, the viral load (*V*) would equate to a proportionality constant (*K*) times the number of short-lived infected CD4 cells (*C*) which would decay at a constant rate (*d)*. Because of assumption three, *C* cannot increase after treatment initiation. In other words, the viral load would be described by the following equation: *V(t) = K C(t*_*0*_*) e*^*-dt*^, where t_0_ is the time of treatment initiation, and t represents the time since *t*_*0*_. This equation can only describe one phase of viral decay. This incoherence inevitably calls into question the above assumptions. We will now show how two of these assumptions have been revisited in order to give rise to two competing hypotheses, one of which is further studied herein.

In particular, the assumption of a single source of plasma virion (short-lived CD4 cells) has been largely disputed regarding its involvement in the slowdown of the viral load decay after the rapid first phase.[1, 7] Other sources of virions have been suggested to explain the occurrence of this second phase decay. One possible explanation put forward is the existence of infected cells that can survive for a longer period (long-lived) (Fig 1B).[1] Each of the four decay phases would correspond to a specific virion-producing cell population.[8] Recent work suggests the long-lived cells responsible for the second phase of decay could integrate viral DNA more slowly, explaining the delay before virion production.[9] These cells could potentially be resting CD4 cells or macrophages, which do exhibit slower integration when investigated *in vitro*.[10, 11] Other authors also mention dendritic cells or monocytes as a potential source of virions explaining the second phase of decay.[12–15] However, uncertainties prevail as to the kinetics of these infected cells and the total viral contribution of these cells *in vivo*. So far, kinetics estimates are solely based on data fitting using models that assume the involvement in viral decay of secondary sources of virions.[9] However, model assumptions can drastically change the values of these estimates since models solely involving cells with a uniform half-life can still fit the viral decay data quite well, as will be later seen.

Recent work challenges the notion that HAART has the capacity to fully inhibit all new cell infections in all organs. Indeed, there can be a large difference in drug concentrations across tissues within the host.[16] For example, one study reports very poor penetration in lymph nodes for all tested antiretrovirals, and several poorly penetrated ileum and rectum tissues.[16] Testicular tissue is another example of a drug sanctuary for many antiretrovirals,[17] along with the central nervous system.[18] The extent of drug penetration in specific secondary lymphoid tissue is largely unknown for most antiretrovirals.[16] Motivated by this and by reported evidence of ongoing viral replication for patients taking HAART,[19–21] we were led to question the role drug penetration may play in explaining the first phases of viral decay, as proposed by Murray et al. [2] The potential involvement of new cell infections in a physiological compartment with limited drug penetration provides an alternative perspective. Under this hypothesis, cells infected before treatment initiation would produce the majority of virions lost during the first phase of decay, while cells infected under treatment within a drug-limited compartment would produce the virions lost during the second phase of decay. Long-lived infected cells would have a negligible impact on viral load, infected cells being short-lived CD4 cells. A depiction of this hypothesis is provided (see Fig 1C).

Better characterizing the involvement of a drug-limited compartment is important, since a compartment harbouring a large quantity of infected cells and with little drug exposure could affect long-term treatment efficacy. Indeed, a large viral population implies a high chance for resistant mutants to be already present before treatment initiation, as the virus naturally mutates at a high frequency.[22] Low drug levels could then lead to suboptimal suppression of mutants that resist to antiretroviral drugs. However, factors such as the decreased viral fitness of resistant strains, compared to wild-type virus, could offset resistant viral growth.[23] Further, concomitant use of multiple drugs could prevent the exponential growth of resistant strains, as such strains may be sufficiently susceptible to one or more of the drugs.

We have developed a mathematical model accounting for these various factors. Our previous mechanistic model describes viral and short-lived infected CD4 cells dynamics in one compartment, namely lymph nodes.[24] The model can explain the long-term risk of virologic failure.[24] Herein, this model was modified to include two compartments, one with low and another with high drug penetration. The compartment with low drug penetration, hereon referred as the drug-limited compartment, partially allows the infection of CD4 cells by the wild-type virus. The compartment is not associated to a specific tissue at this point, as its existence is hypothesized. The compartment with high drug penetration comprises tissues where drug concentrations are sufficient to prevent new cell infection events from wild-type virus (e.g. hypothetically the plasma and well-irrigated tissues).

Our approach was integrative and characteristic of the quantitative pharmacology of systems (QSP).[25] Compared to classical ‘top-down’ approaches that use empirical observations to deduce a mathematical relationship between measured input and output, bottom-up approaches, including QSP, integrate all the information available on the involved processes to build a mechanistic model that describes the hypothesized causal links between input and output. This mechanistic model is then used to predict the output from input values. The produced predictions are compared to empirical observations in order to evaluate if the hypothesized system can explain the phenomenon under investigation.

Our first objective is to evaluate the capacity of the new mechanistic model to predict viral loads observed at the initiation of treatment, particularly the occurrence of two phases, the time delay between phases and the rates of decay. For this purpose, the two-month viral load data of 6 patients taking nelfinavir, zidovudine and lamivudine were fitted.[1] Data was fitted using a least square approach. As a second objective, we examined whether results from this model are consistent with the observed risk of long-term virologic failure. Short- and long-term viral load data of patients taking efavirenz, tenofovir disoproxil fumarate (DF) and emtricitabine combination therapy were used.[26, 27] The former dataset was used to estimate model parameters using a least-square approach, while the latter was used to compare with model predictions. Depending on whether we assess the short- or long-term viral load evolution for treatment naïve patients, resistance mutations are either excluded or included as part of our model, respectively.

## Results

The QSP approach adopted here has previously led to a model that serves as a basis for this study.[24] This previous model, which only looked at infected CD4+ lymphocytes in active state as a source of virions, has been modified in order to simultaneously consider two physiological compartments differently exposed to antiretroviral drugs. One compartment represents the blood compartment and well-irrigated tissues where it is assumed drug penetration is sufficient to prevent new cell infection by wild-type virus. The other compartment is drug-limited. In the following, the former compartment is identified with index 1, while the drug-limited compartment is identified with index 2. The model parameters requiring *a posteriori* estimation are: 1) the average fraction of total infection events not affected by the drugs in each compartment for the wild-type virus (*f*_*u*,*1*_, *f*_*u*,*2*_), and 2) the contribution of each compartment to the maximum plasma viral load or setpoint, in percent (*φ*_1_,*φ*_2_). The values for parameters *f*_*u*,*1*_ and *f*_*u*,*2*_ are mathematically associated with drug concentrations in the respective compartments, with higher drug concentrations leading to smaller *f*_*u*_ values (see Methods and Eq 1 for detail).[28] As for parameters *φ*_1_ and *φ*_2_, an illustrative example would be if one compartment contributes 20% of the viral load when the patient reaches the peak viral load, then the value of *φ* associated with this compartment would be 20%. Since there are only two compartments, the value of *φ* for the other compartment would be 80%.

### Does a model with a drug-limited compartment hosting new short-lived CD4 cell infections have the capacity to predict viral load decay following treatment initiation?

**Fig 2:**
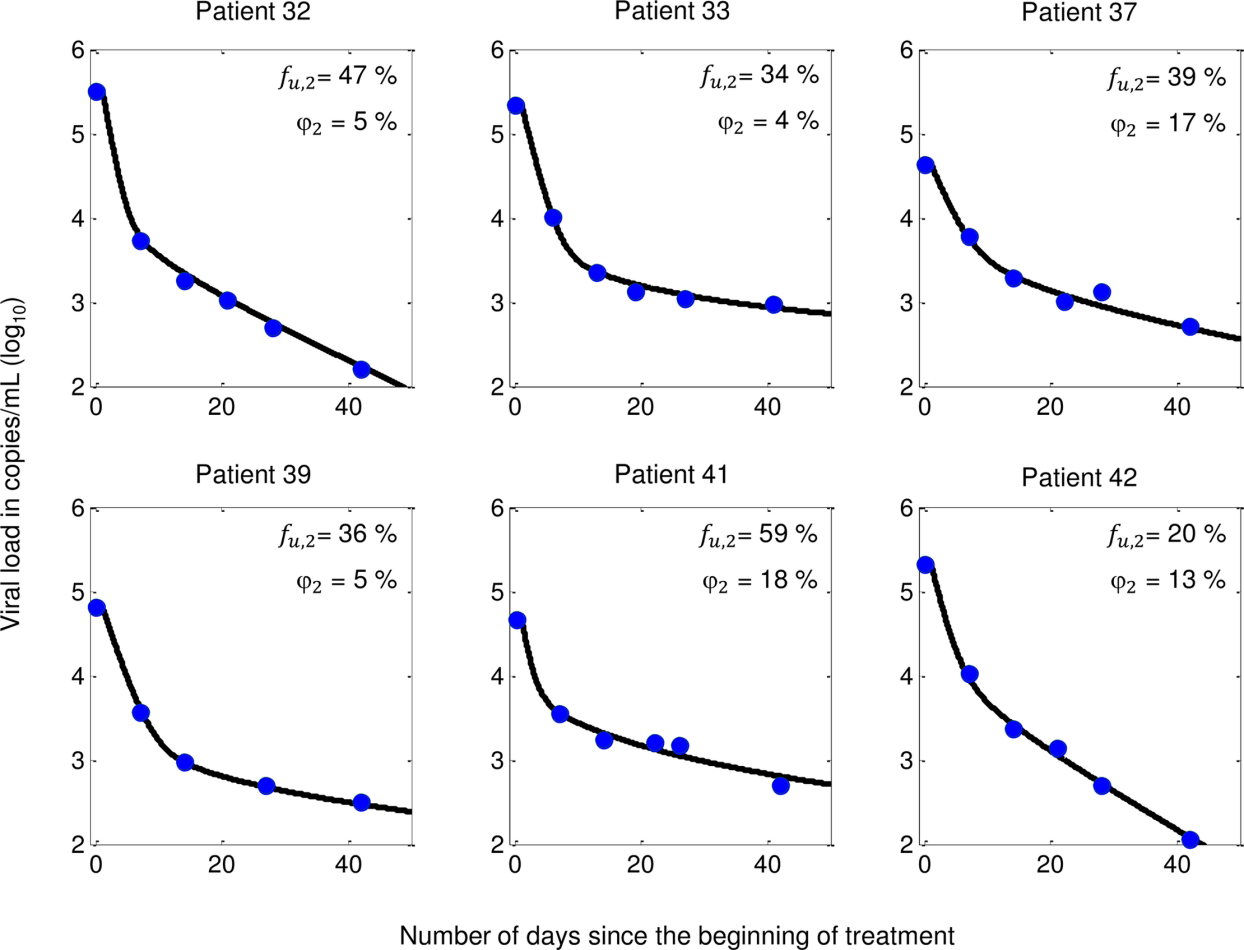
Viral load data extracted from Perelson et al.[1] (blue dots), model fit (black curve), and associated *f*_*u*,*2*_ and *φ*_2_ values. *f*_*u*,*2*_ is the average fraction of total infection events not affected by the drugs and *φ*_2_ is the fraction of the plasma viral load setpoint. Both parameters refer to the drug-limited compartment.

Viral load data were retrieved using WebPlotDigitizer software[29] for the 6 patients reported in Perelson et al.[1], displayed in Fig 2, blue dots. All patients were treatment naïve and initiated a therapy combining three antiretroviral drugs (nelfinavir, zidovudine and lamivudine). Patient demographics for this study can be found in Table 1 of the referenced article.[1]

The short-term viral load decay of the 6 patients can be recovered using our mechanistic model, while keeping the model structure as well as the *a priori* determined parameter values. One of the two compartments is assumed to be sufficiently exposed to medication to prevent further CD4+ infection, i.e. *f*_*u*,1_ = 0. This compartment represents physiological compartments where antiretroviral drug concentrations are sufficiently high to prevent any new cell infection (e.g. hypothetically the plasma and well-irrigated tissues). Results from data fitting procedures for the remaining parameters, using a least-square approach, are shown in Fig 2. With these parameter values, our model predicted the two phases of viral load decay for the 6 patients. Fractional values *φ*_2_ ranging from 4 to 18% allowed the best adjustments. Also, when the drugs inhibit 41% to 80% of the infection in the drug-limited compartment (corresponding to 1-*f*_*u*,*2*_), the obtained predictions are the closest to the viral load observations.

To assess whether the above short-term findings still hold for longer-term virologic response, the model was used to simulate viral load dynamics on a period longer than two months. For this, we first determined a *f*_*u*,*2*_ value using short-term viral load data for patients under efavirenz, tenofovir DF and emtricitabine (600, 300 and 200 mg daily) combination therapy.[26] Secondly, values around the *f*_*u*,*2*_ estimate were translated in terms of drug concentrations in the drug-limited compartment. Finally, these concentrations served to simulate the viral load evolution over a period of approximately one year. This time, resistant strains were allowed to emerge. Virologic failure at 48 weeks was the simulated outcome. To compare the simulation results with clinical data, the virologic failure threshold was set to 400 copies per mL of plasma.[27]

### What values of *f*_*u*,2_ and *φ*_2_ allow reproducing the first two phases of decay in a typical patient under efavirenz, tenofovir DF and emtricitabine?

**Fig 3:**
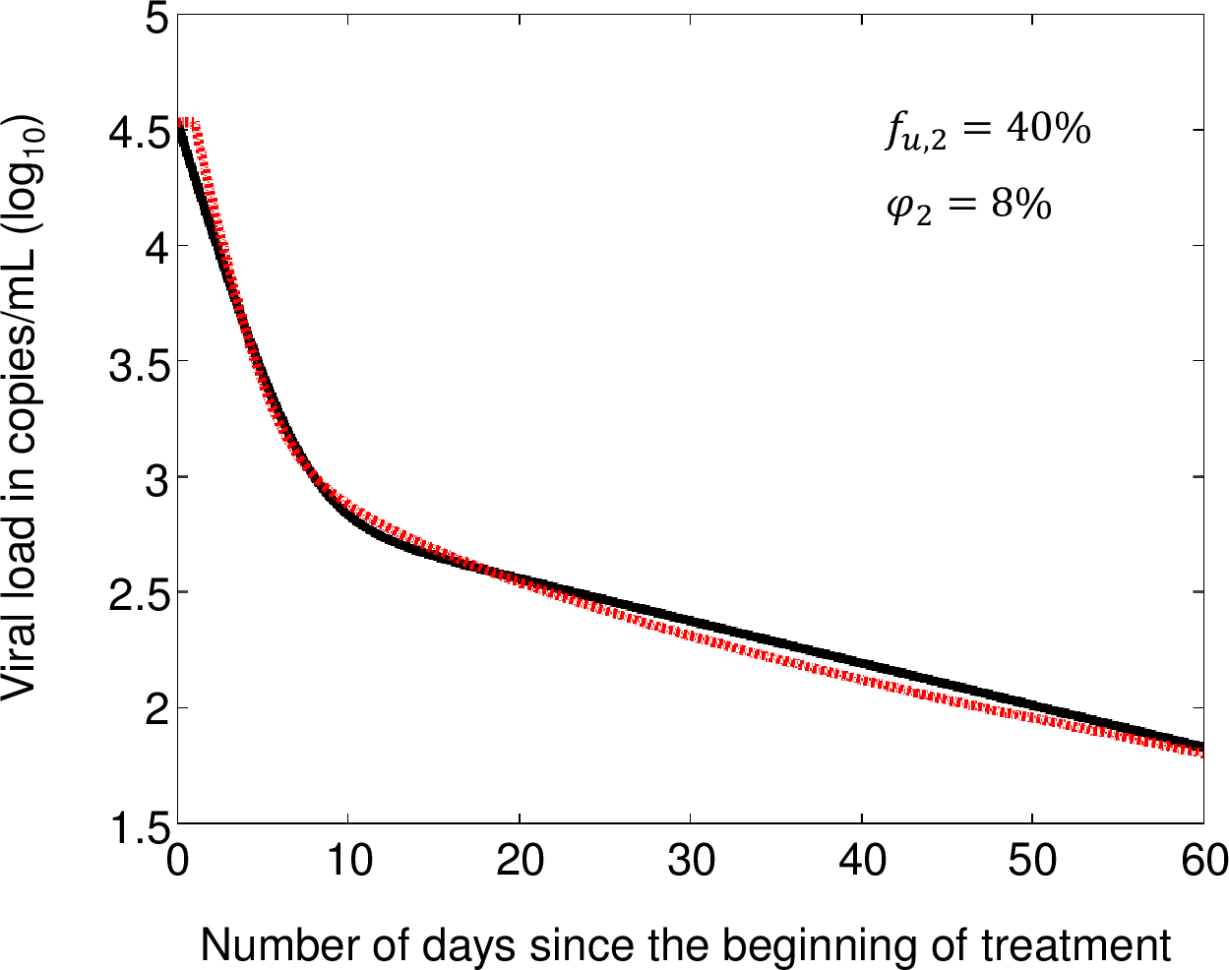
Regression line (black) for the typical patient undertaking a treatment combining efavirenz, tenofovir DF and emtricitabine, model fit (red), and associated *f*_*u*,*2*_ and *φ*_2_ values. *f*_*u*,*2*_ is the average fraction of total infection events not affected by the drugs and *φ*_2_ is the fraction of maximum plasma viral load. Both parameters refer to the drug-limited compartment. Regression curve based on data from Karris et al.[26].

Karris et al. reports a mixed-effect biexponential regression model for the viral loads of 25 patients initiating the combination treatment containing efavirenz, tenofovir DF and emtricitabine.[26] Patient demographics for this dataset can be found in Table 2 of the referenced article.[26] To determine *f*_*u*,2_ values, we focused on the typical patient’s viral loads, in the sense that the patient is characterized by median values of the regression model parameters. In Fig 3, we superimposed the best fitting decay curve predicted by our model on the regression curve representing the viral load decay over a two-month period for the typical patient.[26] The values of *f*_*u*,2_ and *φ*_2_ used in our model were obtained in the same way as done for Fig 2. The values for these parameters allowing the adjustment in Fig 3 are 40% and 8%, respectively. A value of 40% for *f*_*u*,2_ corresponds to an inhibition of 60% of the infection activity in the drug-limited compartment.

### What drug concentrations lead to an inhibition of 60% of the infection in the drug-limited compartment?

The parameter value *f*_*u*,2_ = 40% was translated into concentration values of efavirenz, tenofovir and emtricitabine in the drug-limited compartment. In fact, the association between *f*_*u*,2_ and concentrations were mathematically described for many drugs using *in vitro* experiments.[30] The *in vivo* relationships can be deduced from these associations by assuming that intracellular concentrations dictate drug efficacy, independently of the medium in which the cells reside.[24] The concentration inside the mononuclear cells of a compartment can be derived from extracellular plasma concentrations using two parameters, *k*_*p*_ and *k*_*l*_ (see Eq 1 from Material and Methods). At steady-state, the median extracellular plasma concentrations *C*_*p*_ were extracted from population pharmacokinetics studies.[31–33] Parameter *k*_*p*_, reflecting the effect of plasma protein binding on drug efficacy, is independent of compartmentalization and has been determined empirically elsewhere.[34] Parameter *k*_*l*_, representing the ratio of drug concentrations inside peripheral blood monocyte to the equivalent in the compartment, is the sole unknown value to determine *f*_*u*,2_. When a single drug is used, the relationship between *f*_*u*,2_ and *k*_*l*_ is one-to-one, and the latter can be uniquely determined. When multiple drugs are used concomitantly, the procedure is more complex as the values *k*_*l*_ for each drug need to be determined ($k_{l}^{EFV},\;k_{l}^{TFV}$ and $k_{l}^{FTC}$ for efavirenz, tenofovir and emtricitabine, respectively). Further, different combinations of drug concentrations can lead to the same value of *f*_*u*,2_, after accounting for interaction in drug effects.[35] This translates into multiple combinations of $k_{l}^{EFV},\;k_{l}^{TFV}$ and $k_{l}^{FTC}$ leading to the same value of *f*_*u*,2_. Hence, using a computational iterative procedure, we determined the combinations $k_{l}^{EFV},\;k_{l}^{TFV}$ and $k_{l}^{FTC}$ leading to an inhibition of 60 ± 5% of the infection activity.[24]

**Fig 4:**
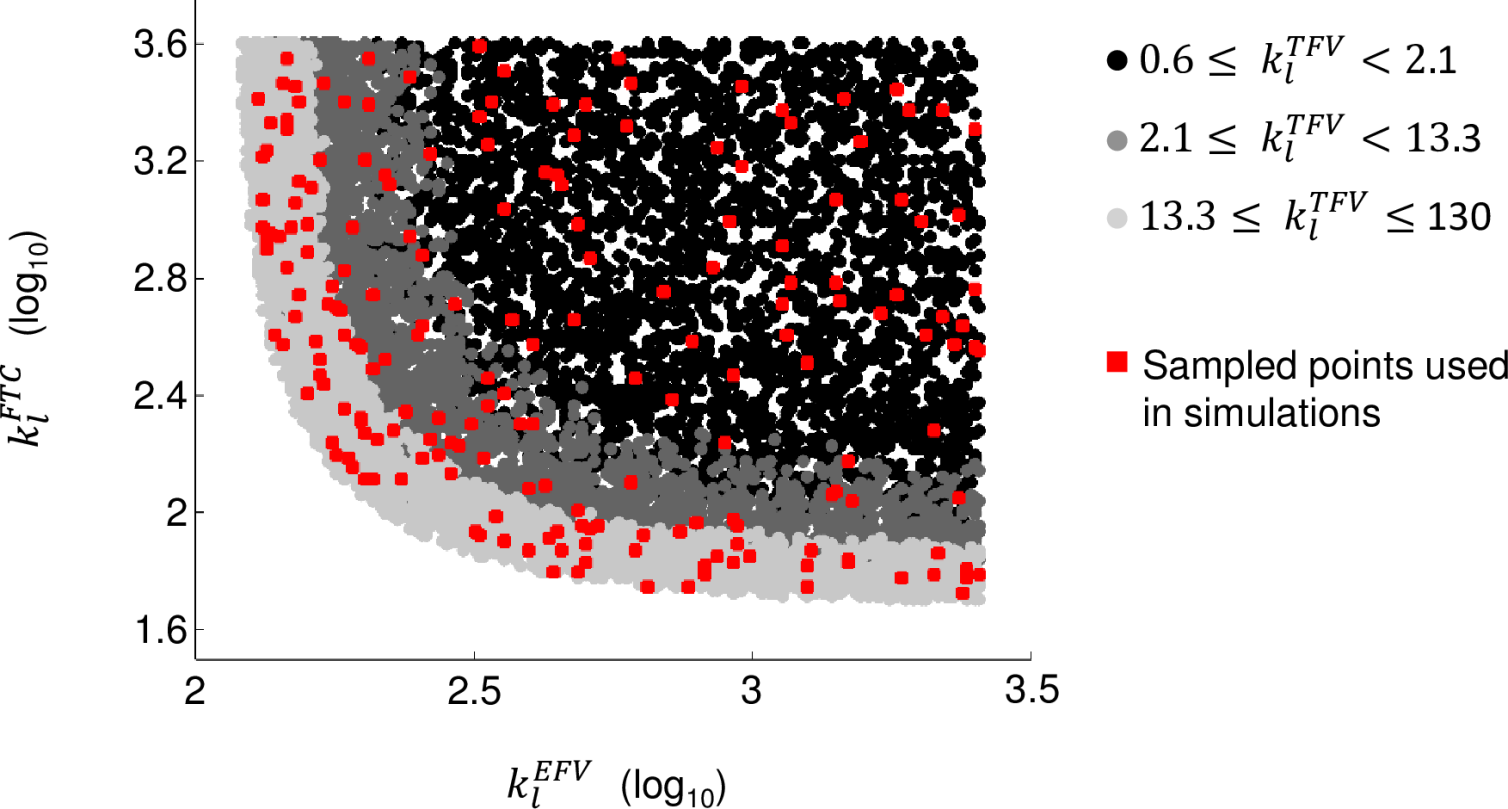
Space of *k*_*l*_ parameter values associated with efavirenz (EFV), tenofovir (TFV) and emtricitabine (FTC) leading to an inhibition of 55%-65% of infection events in the drug-limited compartment. Parameter *k*_*l*_ represents the ratio of concentrations inside peripheral blood mononuclear cells to its equivalent in the hypothesized drug-limited compartment.

The space of parameter values is displayed in Fig 4. In general, a higher *k*_*l*_ value indicates less drug penetration in the hypothesized compartment compared to peripheral blood, while a value of *k*_*l*_ < 1 indicates a higher drug concentration in the compartment. The $k_{l}^{EFV}$value ranges between 88 and 2593, $k_{l}^{TFV}$ between 0.6 and 130 and $k_{l}^{FTC}$ between 32 and 4000. Inguinal lymph nodes are known to be less exposed to efavirenz, tenofovir and emtricitabine.[16] For comparison purposes, we here give the reported values of $k_{l}^{EFV},\;k_{l}^{TFV}$ and $k_{l}^{FTC}$ in this tissue, which are 16.7, 2.9 and 5, respectively, implying a greater drug exposure in lymph nodes as compared to the hypothesized drug-limited compartment.[16]

### What is the expected risk of virologic failure over a long-term period if the less exposed compartment allowing the infection of new short-lived CD4 cells is responsible for the second phase of viral load decay?

The values $k_{l}^{EFV},\;k_{l}^{TFV}$ and $k_{l}^{FTC}$ associated with the drug-limited compartment were used to compute drug efficacy through viral load curves over 48 weeks of therapy with efavirenz, tenofovir DF and emtricitabine (600, 300 and 200mg daily). For this *in silico* experiment, we considered the possibility of the emergence of resistant strains through single nucleotide mutations responsible for the K103N, Y181C, G190S, M184V or K65R substitutions, as used in our previous study.[24] In the simulations, these strains could be generated *de novo* or be selected. We used reported empirical curves of strain-specific drug efficacy and viral fitness.[28, 30] Virtual patients expressed inter-individual variability in pharmacokinetics and immune response, modeled using patient population data.[31–33, 36, 37] This inter-individual variability was added so groups of virtual patients are representative of real patient populations, as in Sanche et al.[24] Due to the uncertainty in drug penetration levels in the second compartment (the *k*_*l*_ values), one simulation was carried out for each of 200 sampled trios of parameter values $k_{l}^{EFV},\;k_{l}^{TDF}$ and $k_{l}^{FTC}$, selected using simple random sampling (points shown in red in Fig 4). For each trio of parameter values, we calculated the proportion of individuals expected to experience virologic failure over 100 simulated virtual patients.

**Fig 5:**
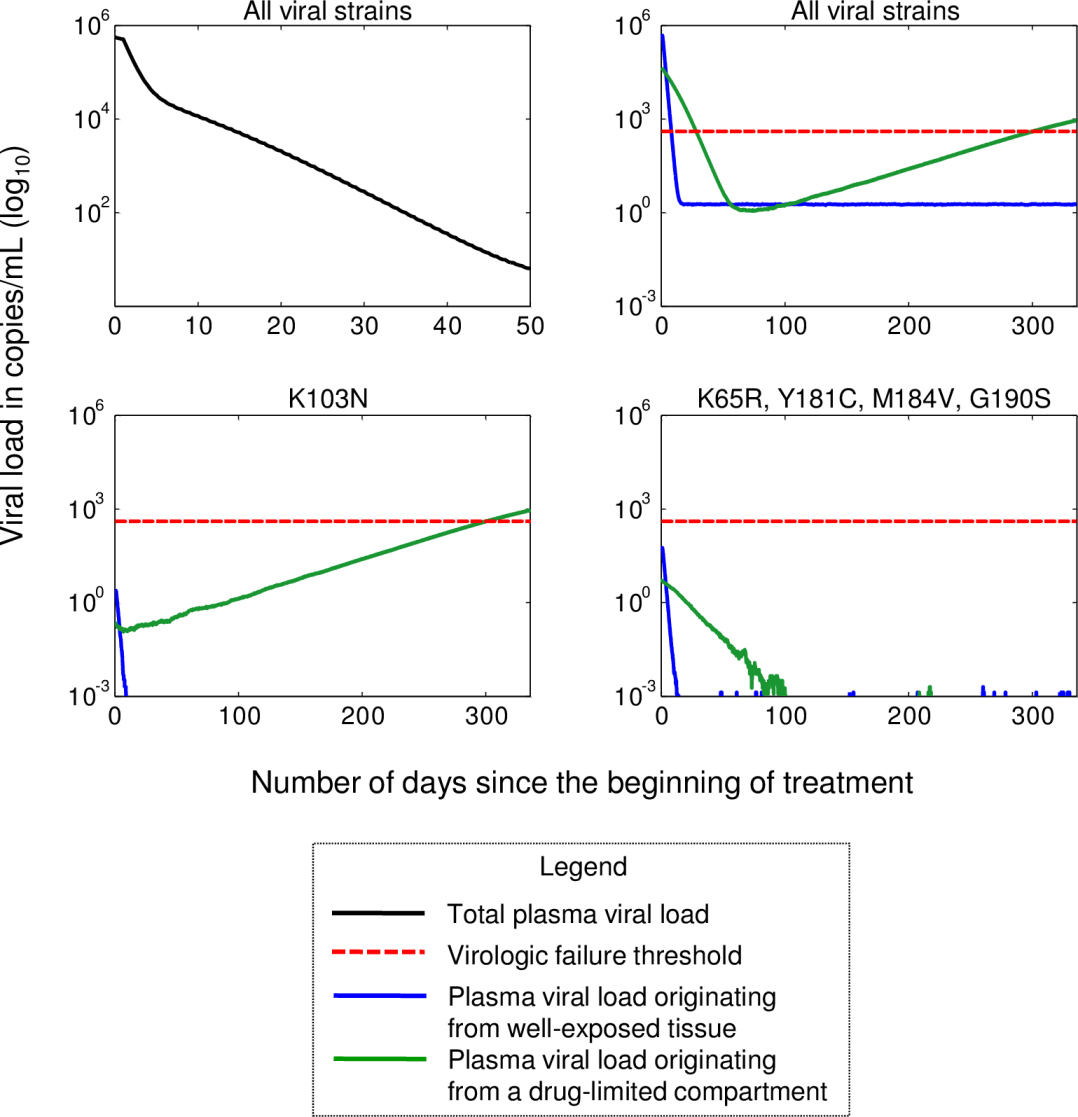
Simulation results for one random patient after 48 weeks of therapy with efavirenz (600mg), tenofovir DF (300mg) and emtricitabine (200mg) taken once daily. The model included a drug-limited compartment that allowed new short-lived cell infections. The virologic failure threshold was set at 400 copies/mL.

To give the reader a better sense of the performed simulations, the predicted viral load of one of the simulated patients, as a function of time since treatment initiation, is shown in Fig 5. The transition from phase one to phase two can be observed approximately a week after treatment initiation (Fig 5, upper left). The long-term viral load originating from each compartment is also presented (Fig 5, upper right). In this example, the K103N mutation was selected in the drug-limited compartment (Fig 5, bottom left). There was no selection for other strains (Fig 5, bottom right).

**Fig 6:**
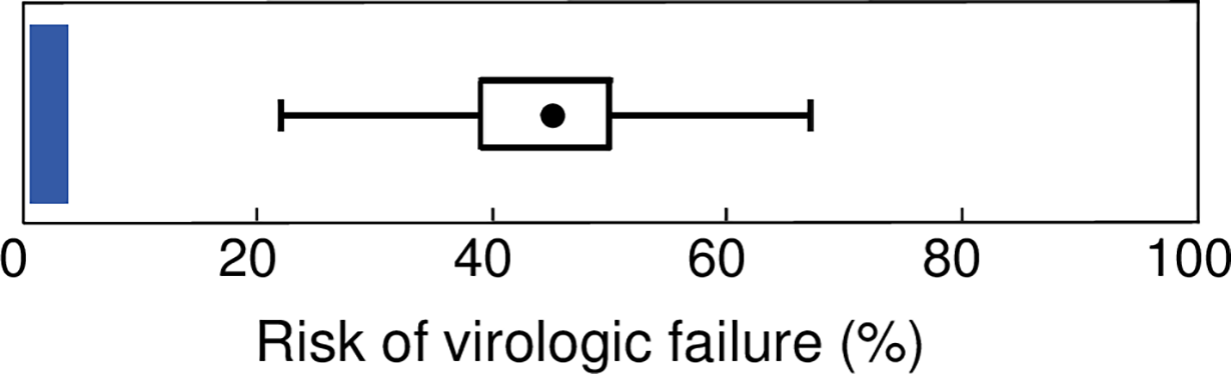
Boxplot of the risk of virologic failure as predicted by the model and obtained from 200 simulations, one per sampling point of drug penetration values (*k*_*l*_^*EFV*^, *k*_*l*_^*TFV*^, *k*_*l*_^*FTC*^). For comparison purposes, the area shaded in darker blue is the confidence interval for the equivalent but observed in a real patient sample.[27].

We used a virologic failure threshold of 400 copies/mL to match the definition used in a previously published clinical study.[27] Patient demographics for this study can be found in Fig 1 of the referenced article.[27] A box plot for the predicted risk of virologic failure was compared to clinical data.[27] As illustrated in Fig 6, we obtained a range of values for the risk of virologic failure going from 22% to 67% at the end of the trials. Most virtual cases of virologic failure were with resistance (>20% of the viral load attributed to resistant viral strains), with K103N and K65R being the most common mutations conferring resistance (data not shown). The smallest value over all 95% confidence intervals for the predicted risk of virologic was 17.4%. In comparison, the risk of virologic failure (>400 copies/mL) based on clinical observation is 2% of patients (4/210 patients), with a 95% confidence interval of [0.5;3.8]%.[27] The p-value for the absence of difference of the proportion of failure between the empirical sample and the model-based sample associated with the smallest predicted risk is smaller than 1E-08. Those who left the clinical study for reasons unrelated to their viral load observations were discarded before computing the empirical risk of failure (pregnancy (n = 4), adverse events (n = 9), lost to follow-up (n = 12), withdrew consent (n = 5), other (n = 4)),[27] explaining the difference with the value reported in the original paper (16% without a response).[27]

We then questioned whether it was possible to reproduce the empirical risk of failure using *φ*_2_ and f_u,2_ values around their estimates from the main analysis (8% and 40%, respectively). For the purpose of this sensitivity analysis, additional simulations were performed by first allowing *φ*_2_ to take values of 6%, then 10%. For each new *φ*_2_ value, we sampled 50 points in the space of parameter values for $k_{l}^{EFV},\;k_{l}^{TFV}$ and $k_{l}^{FTC}$ associated with an unchanged f_u,2_ and, for each point, computed the risk of failure over simulations of N = 100 virtual patients. Predicted probabilities of failure fell within the range obtained in the main analysis (22% to 67%). Using a similar approach, we computed the risk of failure assuming f_u,2_ values of 30%, then 50%, with an unchanged *φ*_2_. The smallest risk of failure over these simulations was 17% (95%CI [10.2;25.8]%). We further extended our analysis to f_u,2_ values of 20% and 60%. For these parameter values, the smallest predicted risk of failure was 9% (95%CI [4.2;16.4]%) and was achieved for a f_u,2_ value of 20%. Under this f_u,2_ value, the slope of second phase decay for the typical individual would be more than 2.5 times greater than its empirical estimate.

## Discussion

The viral load of most HIV-infected patients initiating HAART decays in distinct phases.[4] During each phase, the viral load decays at an approximately constant rate. However, this rate decreases substantially from one phase to the next. During the final phase, the decay is so slow that low-level viremia (<50 copies/mL) persists over many years of therapy.[38] To date, the causes behind these phases, which characterize the viral decay profiles, still draw attention and fuel a debate. One hypothesis advances that the second phase of viral decay would be caused by infected cells that could survive longer than short-lived CD4 cells.[1] Evidence suggests these cells could be CD4 cells exhibiting a slower integration of viral DNA.[9] Another hypothesis stipulates that new short-lived infected cells from a drug-limited compartment could cause the phenomenon.[2] We investigated the latter hypothesis.

Contrary to the role long-lived cells could play in this phenomenon, much less attention has been paid to the involvement of drug penetration in tissues. Evidence for the existence of tissues where new cell infections occur during therapy,[19–21] and suggesting high variability in drug penetration in terms of specific lymphoid tissue,[16] adds elements of controversy to the ongoing debate. In the current work, we focus on the first two phases of viral decay without the specific differentiation between the sub-phases 1a and 1b that have been recently revealed.[9, 39] With the objective to shed additional light on the existence of multi-phasic viral load decay, we here compared predictions from a mathematical model to short-term viral load data as well as to long-term clinical outcomes.

We overlaid our model predictions on short-term viral load data of 6 patients. *De visu*, the agreement between model predictions and patient observations is substantial (Fig 2), and compares with the results obtained through a model assuming short- and long-lived virion-producing cells.[1] This suggests both models are plausible mathematical explanations for the short-term viral load decay after treatment initiation. In fact, the results indicate that in order *t*o reproduce the short-term viral loads, the infection activity in the less drug-exposed compartment has to contribute from 4% to 18% of the total plasma viral load before treatment initiation. Further, the drugs in this compartment would need to prevent 41% to 80% of all new cell infections for the model to accurately fit the data. This suggests the associated tissues need to harbour a substantial amount of the total infection activity before treatment initiation (infection hot spot). Moreover, the level of drug exposure would likely be much smaller in this compartment than what is prohibitive in infection assays.[30]

The existence of a drug-limited compartment allowing new short-lived CD4 infections could affect long-term drug resistance. To study this, we based ourselves on a model able to reproduce the observed risk of virologic as a function of the adherence level of a population of patients initiating treatment with efavirenz, tenofovir DF and emtricitabine.[24] This model accurately predicted the risk of virologic failure observed in patients after long-term therapy (1 to 1.5 years) under various regimen and drug adherence.[24] Herein, this model was modified to consider two compartments differentially exposed to drugs. The principles governing viral kinetics in the compartments were kept: both models used an *in vivo* extrapolation of drug susceptibility assays along with empirical data on *in vivo* antiviral activity.[24] Further, the extent of the immune response or whether resistant virus is present when treatment starts, which may also impact viral growth, are equivalently considered in both models.[24]

We used the modified model to simulate the viral activity within a population of patients initiating treatment with efavirenz, tenofovir DF and emtricitabine. Here, we first deduced that even if 100% infections are prevented in well-irrigated tissues, 60% of wild-type virus infections have to be inhibited in a second compartment to explain the first two phases of decays observed in patients (Fig 3, *f*_*u*,2_ = 40%). This value is consistent with what was obtained in Fig 2. Second, we determined the drug concentrations within this compartment corresponding to such level of inhibition. Finally, we used these concentrations to simulate long-term viral suppression in small groups of patients. To palliate the uncertainty in the combination of drug concentrations leading to 60% inhibition of cell infection events, we undertook 200 simulations, one per selected trio of drug penetration parameters $k_{l}^{EFV},\;k_{l}^{TFV}$ and $k_{l}^{FTC}$. We used a simple random sample, using the log scale of *k*_*l*_ values, to obtain the sampled values in red in Fig 4. The chosen points are well spread across the domain of allowable values, indicating a good representation of the entire space. Results in Fig 6 suggest a minimum of 22% of patients would experience virologic failure at the end of 48 weeks of treatment under the evaluated hypothesis (lower bound of 95% CI of 17.4%). The predicted risk is not consistent with the 2% risk of virologic failure reported in a clinical study we used to validate our model (95% confidence interval of empirical virologic is [0.5%;3.8%]).(21) Sensitivity analyses also showed this inconsistency remains once uncertainty in parameter values has been accounted for. Even if the median slope of second phase viral decay was 2.5 times its empirical estimate (f_u,2_~20%), it was still impossible to achieve overlap between predicted and empirical confidence intervals of the risk of failure.

The mechanistic nature of our model enables evaluating the likelihood of the hypothesis of new short-lived CD4 cell infections in a drug-limited compartment explaining the second phase of viral decay.[25] The model consists in the physiological, pharmacological and viral elements interacting to dictate the evolution of the viral population within its host. While there is an increasing body of evidence suggesting the existence of drug-limited tissues impacting the viral load dynamics, such as lymph nodes,[16] the results of the current study suggest persistent short-lived CD4 infections in a drug-limited compartment cannot–by itself–explain the second phase of viral decay. Here, all parameter values, with the exception of the ones that are linked to the hypothesis in question (*φ*_2_ and *f*_*u*,2_), were determined *a priori* from reported experiments. Plasma drug concentrations were simulated using models derived from population pharmacokinetic studies.[31–33] The relationship between drug concentrations and drug effect on each separated strain was based on an *in vitro* to *in vivo* extrapolation of drug efficacy.[16, 28, 30, 34, 35] The within-host growth rate of viral loads was also based on reported distributions.[36, 37] Finally, the probability of emergence of specific mutations as well as the fitness of each strain were well described in the literature.[28] However, it was not possible to find parameter values consistent with the hypothesis and allowing coherent predictions of long-term virologic responses.

The model we formulated can suggest other explanations for the second phase of viral load decay. Because mass action law dictates the dynamics of the infection in the model,[40] how quickly the number of infected cells grows or declines over time is dependent on the availability of target cells. A lower density of target cells in a compartment means that virions have a higher chance to be cleared before they can infect a cell, which leads to a decline in the infection rate while the decay rate of infected cells remains the same. When the density of target cells is too low, the model predicts a constant decline of virion production over time with a rate dependent on density. In fact, we reproduced two phases of viral decay with our model when new cell infections are completely prevented by the drug in one compartment, while in another compartment unexposed to drugs, virion production slowly declines due to the loss of target cells (data not shown). Because of the absence of drug exposure in the second compartment, no long-term selection of resistant mutants occurs.

In reality, a reduction in the number of target cells could be initiated by immune contraction.[41] It is hypothesized that this phenomenon, which implies a rapid decay of effector T cells, can be triggered by a reduction in antigen availability.[41] We hypothesize that one explanation for the first 2 phases of decay could be that the sharp decrease in the density of virions in one compartment that is highly exposed to drugs triggers a contraction phase. This contraction phase, associated with systemic cytokine signaling,[41] could promote the death or inactivation of CD4 cells in another compartment unexposed to drugs.

In the current model, the effect of the immune response is represented via a single parameter that summarizes the growth rate of viral infection in absence of drug action (reproduction number).[24] This is a consequence of a lack of quantitative knowledge about changes in patients’ immunity from disease onset. In particular, little is known about the evolution of the density of activated CD4 cells in infected tissues. Although the fraction of cells expressing CD38+HLA-DR+ among CD4 cells is significantly reduced in the blood of patients after treatment initiation,[42] more precise estimates of local changes in various tissues could lead to a better understanding of the interaction between immunity and the virus.

In summary, the specific cause for the phases of viral decay is still uncertain. On the one hand, the hypothesis advancing the existence of infected cells exhibiting a decay dynamic that is different from short-lived CD4 cells has literature support.[1, 9] On the other hand, the hypothesis stipulating that such cells have a negligible impact on virion production and completely attributing the slower decay of virion production to new short-lived cell infections in a drug limited compartment finds long term inconsistencies. Alternative models that would measure the contribution of both poor drug exposure in tissues and the immune response remain to be studied. A better understanding of the dynamics of activated immune cells before and after treatment is initiated could shed light on the matter.

## Materials and methods

### The model and simulations

An overview of the model is given below. It includes the description of the algorithm and main parameters, as well as the discussion of the source of parameter values. The model is adapted from Sanche et al.[24], the main difference being the consideration of two compartments with different exposures to the drugs.

The model is implemented using an algorithm which iteratively computes the number of events involving active CD4 cells during small time intervals: i) the number of virion-producing cells that die out, ii) the number of new cell infections, and iii) the fraction of these infections that involve a newly mutated virus. Step i) is computed using a constant cell death rate (*d*_*y*_). Step ii) is computed from reproduction numbers $R_{i}^{j}$, where *i* and *j* indicate the strain of the virus and the compartment, respectively. Details on reproduction numbers are given below. The fraction of *de novo* mutation is based on probabilities of SNP mutations estimated from empirical data and reported elsewhere.[43] Total infection activity is translated in plasma virions, by assuming one virion-producing cell supplies one plasma virion, a relationship that is consistent with independent data.[5, 44]

The model assumes compartments are isolated from each other in terms of viral infection, i.e. a virus produced by one cell can only infect cells within the same compartment. This has been confirmed with experimental data: the infection of new cells is considered an essentially local phenomenon and the genetic makeup of the viral populations suggests a high degree of compartmentalization.[21, 45] The modeled viral dynamics is very similar in each compartment, the main difference being the level of drug exposure influencing the reproduction numbers.

Reproduction numbers $R_{i}^{j}$ are expressed as *R*_*0*_ (1-*s*_*i*_) $f_{u,j}^{i}$, where *R*_*0*_ is the mean number of CD4 cells becoming infected by viruses produced by a single infected cell when susceptible cells are abundant and when no drug is present, *s*_*i*_ is a fitness cost for strain *i*, and $f_{u,j}^{i}$ is the fraction of CD4 infection events unaffected by the drugs for strain *i* in compartment *j*. In this study, $f_{u,j}^{i}$ was either estimated for wild-type virus from viral load data (index *i* is omitted in this case, since only wild-type virus was considered), or computed from drug concentrations. In the latter case, plasma drug concentrations are first simulated from reported pharmacokinetic models. Since the concentrations vary over time *t*, so do the $f_{u,j}^{i}$. In the particular case where only one drug is used, $f_{u,j}^{i}$ takes a relatively simple form (Eq 1):

$$f_{u,j}^{i}(t)=\frac{1}{1+{\left(\frac{C_{p}(t)}{{\rho_{i}IC}_{50}^{0}k_{p}k_{l,j}}\right)}^{m(1+\sigma_{i})}}$$

where *C*_*p*_(*t*) is the plasma drug concentration at time t, *k*_*p*_ is the coefficient adjusting for plasma protein binding, *k*_*l*,*j*_ is the coefficient adjusting for the degree of drug penetration in the compartment *j*, respectively, ${IC}_{50}^{0}$ is the concentration inhibiting 50% of infection events by the wild-type strain *in vitro* in a medium devoid of plasma proteins, *m* is the Hill coefficient, and finally *ρ*_*i*_ and *σ*_*i*_ are two factors adjusting the values of ${IC}_{50}^{0}$ and m, respectively, for the resistant viral strain *i*. For the wild-type strain, *ρ*_*i*_ = 1 and*σ*_*i*_ = 0. It should be noted that to estimate the impact of concomitant drug use, $f_{a,j}^{i}(t)$ no longer takes a simple analytical form (see Jilek et al. and Sanche et al.).[24, 35]

Parameters φ_j_ are used in all simulations to limit the number of cells each compartment may contain. The overall number of cells is itself dictated by parameter λ, which is an entry rate of uninfected activated CD4 cells.

Many parameter values are patient-specific. The death rate *d*_*y*_ is randomly selected for each patient from a distribution of values based on empirical measures of the first phase decay.[46] The distribution has a median value corresponding to a half-life of about 0.7 day. The same applies for λ (directly linked to the distribution of viral set points)[28] and *R*_*0*_ (based on the growth rate of viral loads during rebounds).[36, 37] Inter-individual variability for pharmacokinetic parameters is based on the reported values of population pharmacokinetics models.[31, 33, 34, 47] All other model parameters have *a priori* assigned values reported in Sanche et al.[24], with the exception of φ_j_ and *f*_*u*,*j*_.

### Viral load data fitting

Using the developed mechanistic model, data fitting refers to finding model parameters φ_2_ and *f*_*u*,2_ for which viral load predictions are closest to empirical observations. The process was carried out in two steps to reduce numerical calculations. In the first step, we generated combinations of φ_2_ and *f*_*u*,2_ taking values between 5% and 95% in 5% increments (361 combinations). For each combination, the viral loads of 20 virtual patients were simulated. Values of patient-specific parameters *d*_*y*_, λ and *R*_*0*_ were randomly selected from their corresponding patient population distributions. Overall, the viral loads of a total of 7,220 virtual patients were simulated. For each set of real viral load data, we computed the sum of the squared difference between predictions and observations, using a logarithm scale for viral loads, in order to identify the virtual patient with the closest viral load predictions. This numerical experiment was repeated for values of φ_2_ and *f*_*u*,2_ around those retained for the identified virtual patient, noted $\varphi_{2}^{\prime }\;and\;f_{u,2}^{\prime }$. More precisely, all discrete percentage values of *φ*_2_ in $\varphi_{2}^{\prime }$ ± 4% and *f*_*u*,2_ in $f_{u,2}^{\prime }$ ± 4% were investigated (81 combinations, 1620 virtual patients). We report the virtual patient’s viral load being the closest to the observations, using the same criteria as above.

### Identification of $k_{l}^{EFV},\;k_{l}^{TFV}\;\mathrm{and}\;k_{l}^{FTC}$ in the less drug-exposed compartment

Multiple trios of values $k_{l}^{EFV},\;k_{l}^{TFV}$ and $k_{l}^{FTC}$ can lead to the same level of drug efficacy under combination treatment. We identified the space of parameter values for $k_{l}^{EFV},\;k_{l}^{TFV}$ and $k_{l}^{FTC}$ for which *f*_*u*,2_ was within 5% of a target value $\hat{f_{u,2}}$. This was done in an iterative fashion, by exploring a wider space of parameter values $\left[{\;k}_{l,min}^{EFV};\;k_{l,max}^{EFV}]\times [k_{l,min}^{TDF};\;k_{l,max}^{TFV}]\times [k_{l,min}^{FTC};\;k_{l,max}^{FTC}\right]$.

We used Eq 1 to identify the minimum and maximum values of *k*_*l*_ for each drug. The rationale for this procedure was the following. First, we found *k*_*l*,*min*_ such that if *k*_*l*_ < *k*_*l*,*min*_ for each of the three considered drugs, then the value of *f*_*u*,2_ for the combined drug effect will necessarily be beneath $\hat{f_{u,2}}-5\%$. In other words, the use of a single drug would be more than sufficient to inhibit 1-*f*_*u*,2_ infection events in the less exposed compartment. Further, we found *k*_*l*,*max*_ for which if *k*_*l*_ > *k*_*l*,*max*_ implies almost no effect from the drug (too small drug concentration in the tissue to have an effect). More precisely, we computed the ratios *C*_*p*_(*t*)/*k*_*l*_ that correspond to $f_{u,2}=\hat{f_{u,2}}-10\%$ and *f*_*u*,2_ = 99%, where the values for *m*, *k*_*p*_ and ${IC}_{50}^{0}$ are reported elsewhere.[24, 30, 34] We then derived *k*_*l*,*min*_ and *k*_*l*,*max*_ using average steady-state plasma concentrations *C*_*p*_(*t*) (2.54mg/L for efavirenz, 0.183mg/L for tenofovir and 0.55mg/L for emtricitabine).[31–33] Finally, we discretized the range of values for *k*_*l*_ for each of the three drugs using 200 points between *k*_*l*,*min*_ and *k*_*l*,*max*_, leading to 8,000,000, or 200^3^ determined trio values. For each of these trios, we could compute the expected value of *f*_*u*,2_ for the drug combination, by using a procedure further accounting for the degree of independence of drug effects.[24, 35] We retained the subset of ${(k}_{l}^{EFV},\;k_{l}^{TFV},\;k_{l}^{FTC})$ for which the expected value $f_{u,2}\in [\hat{f_{u,2}}-5\%;\;\hat{f_{u,2}}+5\%]$.

### Statistical analysis

Parameter estimation was performed to fit our model to short term empirical data. As explained in the section entitled Data fitting for viral load data, this procedure was based on the least square difference between model predictions and empirical viral load observations in log_10_ scale. The goodness of fit of the model was evaluated visually. We computed the 95% confidence intervals for the risk of long-term virologic using the exact method, both for the empirical and the smallest predicted risks.[48] We also computed the p-value associated to a null hypothesis of equal risk of failure in both the empirical and the model-based samples and assuming the difference of proportion is normally distributed (central limit theorem).[48]
